# ERp57 modulates STAT3 activity in radioresistant laryngeal cancer cells and serves as a prognostic marker for laryngeal cancer

**DOI:** 10.18632/oncotarget.3042

**Published:** 2015-01-08

**Authors:** Min Ho Choe, Joong Won Min, Hong Bae Jeon, Dong-Hyung Cho, Jeong Su Oh, Hyun Gyu Lee, Sang-Gu Hwang, Sungkwan An, Young-Hoon Han, Jae-Sung Kim

**Affiliations:** ^1^ Division of Radiation Cancer Research, Korea Institute of Radiological and Medical Sciences, Seoul, Korea; ^2^ Biomedical Research Institute, MEDIPOST Co., Ltd., Seoul, Korea; ^3^ Graduate School of East-West Medical Science, Kyung Hee University, Suwon, Korea; ^4^ Department of Genetic Engineering, Sungkyunkwan University, Suwon, Korea; ^5^ Department of Microbiology and Immunology, College of Medicine, Yonsei University, Seoul, Korea; ^6^ Molecular-Targeted Drug Research Center and Korea Institute for Skin and Clinical Sciences, Konkuk University, Seoul, Korea

**Keywords:** ERp57, STAT3, Mcl-1, Laryngeal cancer, Radioresistance

## Abstract

Although targeting radioresistant tumor cells is essential for enhancing the efficacy of radiotherapy, the signals activated in resistant tumors are still unclear. This study shows that ERp57 contributes to radioresistance of laryngeal cancer by activating STAT3. Increased ERp57 was associated with the radioresistant phenotype of laryngeal cancer cells. Interestingly, increased interaction between ERp57 and STAT3 was observed in radioresistant cells, compared to the control cells. This physical complex is required for the activation of STAT3 in the radioresistant cells. Among STAT3-regulatory genes, Mcl-1 was predominantly regulated by ERp57. Inhibition of STAT3 activity with a chemical inhibitor or siRNA-mediated depletion of Mcl-1 sensitized radioresistant cells to irradiation, suggesting that the ERp57-STAT3-Mcl-1 axis regulates radioresistance of laryngeal cancer cells. Furthermore, we observed a positive correlation between ERp57 and phosphorylated STAT3 or Mcl-1 and *in vivo* interactions between ERp57 and STAT3 in human laryngeal cancer. Importantly, we also found that increased ERp57-STAT3 complex was associated with poor prognosis in human laryngeal cancer, indicating the prognostic role of ERp57-STAT3 regulation. Overall, our data suggest that ERp57-STAT3 regulation functions in radioresistance of laryngeal cancer, and targeting the ERp57-STAT3 pathway might be important for enhancing the efficacy of radiotherapy in human laryngeal cancer.

## INTRODUCTION

Radiotherapy is the standard treatment modality for localized laryngeal cancer, which is the largest subgroup of head and neck cancer. Radiotherapy is generally preferred in patients with laryngeal cancer because it allows for better organ preservation [1, 2]. Despite progress in the delivery of the ionizing radiation doses over the past 2–3 decades, many patients still suffer from local recurrence after radiotherapy [3, 4]. The survival of a small fraction of radioresistant tumor cells during the course of radiotherapy is the main obstacle for successful radiotherapy. Thus, drugs that inhibit the molecular targets contributing to the radioresistance of tumor cells will be essential to improve radiotherapy. Although several molecular targets modulating tumor survival and the microenvironment have been shown to influence the outcome of radiotherapy [5], clinically relevant targets and signaling pathways are still unclear.

Signal transducer and activator of transcription 3 (STAT3) is a cytoplasmic transcription factor that transmits oncogenic signals from cytokines and growth-factor receptors to the nucleus [6]. Hyperactivation of STAT3 in response to the aberrant activation of upstream receptor signals is frequently observed in a variety of human cancers, including head and neck cancer [6-8]. Persistent STAT3 activation promotes the growth and survival of tumor cells through modulation of cell cycle regulators (e.g., cyclin D1/D2 and c-Myc), upregulation of anti-apoptotic proteins (e.g., Mcl-1, Bcl-xl, and survivin), downregulation of the tumor suppressor p53, and induction of angiogenesis by vascular endothelial growth factor (VEGF); these mechanisms eventually contribute to tumor progression and resistance to anti-cancer drugs [6, 8, 9]. Accumulating evidences indicate that inhibition of STAT3 enhances radiation sensitivity in various tumor cells [10, 11]. In addition, recent reports indicate that JAK/STAT signaling contributes to tumor resistance by modulating not only cell survival but also the tumor microenvironment, including tumor hypoxia and immunity [12, 13]. Thus, targeting STAT3 activation is essential for overcoming tumor resistance to chemotherapy and radiotherapy.

ERp57, also known as GRP58/PDIA3, belongs to the family of protein disulfide isomerases, is recognized as a multifunctional chaperone that regulates proper folding and quality control of glycoproteins, and participates in the assembly of major histocompatibility complex class 1 in the endoplasmic reticulum (ER) [14]. Although ERp57 has been characterized by its functions in the ER, many evidences indicate that ERp57 is also involved in a variety of functions in the cytosol and nucleus [14]. Several reports have suggested that ERp57 is associated with tumor progression and modulation of STAT3 activity [15, 16], although their findings are controversial [17]. For instance, some studies have shown that overexpressed ERp57 is associated with oncogenic transformation in normal rat kidney cells and cellular invasiveness in cervical and breast cancer [18-20], whereas another group showed that loss of ERp57 expression correlates with more aggressive gastric cancer [21]. In addition, ERp57 has been reported to interact with the STAT3 complex, enhancing STAT3 activity in melanoma and hepatoma cells [15, 16], whereas another group suggested that this ERp57-STAT3 complex negatively affects STAT3 DNA-binding activity [17]. Hence, the regulatory roles of ERp57 in tumor progression and STAT3 activity are still undefined.

Previously, we identified ERp57 as an upregulated protein in radioresistant HEp-2 laryngeal cancer cells by comparative proteomic analysis [22]. Here, we further investigated the role of ERp57 in tumor radioresistance through modulation of STAT3 activity in laryngeal cancer. Our data suggest that ERp57 contributes to radioresistance of laryngeal cancer cells by activating the STAT3-Mcl-1 pathway, and this regulation is associated with poor prognosis in laryngeal cancer.

## RESULTS

### ERp57-regulated radioresistance of laryngeal cancer cells

**Figure 1:**
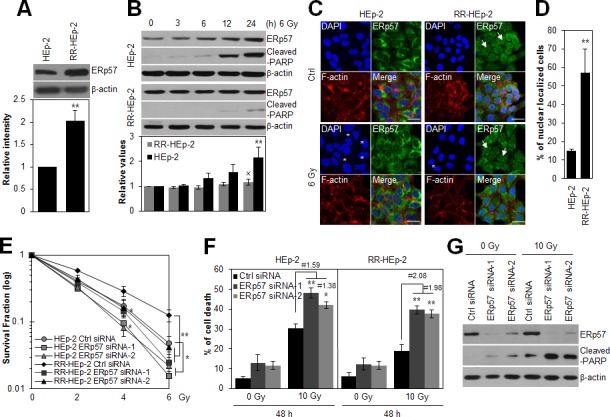
Depletion of ERp57 sensitizes radioresistant HEp-2 cells (A) Lysates of control HEp-2 and RR-HEp-2 cells were immunoblotted with an anti-ERp57 antibody. (B) Control HEp-2 and RR-HEp-2 cells were treated with 6 Gy radiation and cultured for the indicated periods. Expression levels of ERp57 were quantified using ImageJ software from 3 independent experiments (lower panel, A and B). **P < 0.01 compared with HEp-2 (A) or untreated HEp-2 cells (B) and × denotes no significance compared with untreated RR-HEp-2 cells (B). (C and D) HEp-2 or RR-HEp-2 cells untreated (Ctrl) or treated with 6 Gy radiation for 24 h were stained with anti-ERp57 antibody (green), Alexa 568 phalloidin (red), and DAPI (blue). The asterisks and arrows indicate fragmented nuclei and ERp57 nuclear localization, respectively (C). Nuclear-localized ERp57 in HEp-2 and RR-HEp-2 cells was quantified using CellProfiler software (≥100 cells for each dataset). *n* = 3; **P < 0.01 compared with HEp-2 cells (D). (E–G) RR-HEp-2 cells were transfected with 100 nM of control siRNA, ERp57 siRNA-1, or ERp57 siRNA-2, respectively. After 48 h, the cells were treated with each dose of radiation, as indicated, (E) or 10 Gy radiation (F and G). # indicates the ratio for % of cell death of irradiated siRNA control cells / % of cell death of irradiated ERp57-depleted cells. The clonogenic survival fraction was determined by clonogenic assay (E). Cell viability was determined via FACScan flow cytometry, and data are presented as the percentage of PI-positive cells (F). Cells were analyzed by immunoblotting with anti-cleaved PARP and anti-ERp57 antibodies (G). β-actin was used as loading control. The data represent typical results and are presented as mean ± standard deviation of 3 independent experiments; *P < 0.05 or **P < 0.01 compared with irradiated siRNA control cells, respectively (E and F).

ERp57 is upregulated in radioresistant laryngeal cancer HEp-2 (RR-HEp-2) cells and two other laryngeal cancer SNU899 and SNU1076 cells, which are more radioresistant than the control HEp-2 cells (Fig. 1A, Supplementary Fig. 1A and B). Therefore, we hypothesized that upregulated ERp57 possibly associates with the radioresistant phenotype of laryngeal cancer cells. To investigate the role of ERp57 in radioresistance, we first examined the expression pattern of ERp57 in response to irradiation in control HEp-2 and RR-HEp-2 cells. As shown in Fig 1B, ERp57 was upregulated in the control cells in response to irradiation, whereas its expression was unchanged in RR-HEp-2 cells, suggesting that the differential expression pattern of ERp57 is involved in radioresistance of laryngeal cancer cells. In addition, the cellular localization of ERp57 was examined in response to irradiation in HEp-2 and RR-HEp-2 cells because colocalization of ERp57 and calreticulin in the plasma membrane is known to be involved in immunogenic cell death during chemotherapy [23]. Interestingly, ERp57 did not translocate to the plasma membrane in response to irradiation (Fig. 1C), whereas ERp57 was detected in the nuclei of RR-HEp-2 cells but not in the nuclei of control cells (Fig. 1C and D), implying that the nuclear function of ERp57 is associated with the radioresistance. Next, we synthesized two different siRNAs targeting different regions of the gene to further determine the role of ERp57 in tumor radioresistance. Depletion of ERp57 by both siRNAs significantly decreased the survival of HEp-2 and RR-HEp-2 cells in response to various doses of radiation (Fig. 1E) and increased radiation-induced cell death compared to the control, irradiated cells (Fig. 1F and G). Interestingly, ERp57-depleted RR-HEp-2 cells are more radiosensitive than ERp57-depleted HEp-2 cells (Fig. 1E and F). We further confirmed this result in two other laryngeal cancer cells (Supplementary Fig. 1C and D). Taken together, our data suggest that ERp57 potentiates radioresistance of laryngeal cancer cells.

### Increased interaction between ERp57 and STAT3 was associated with radioresistance of laryngeal cancer cells

**Figure 2:**
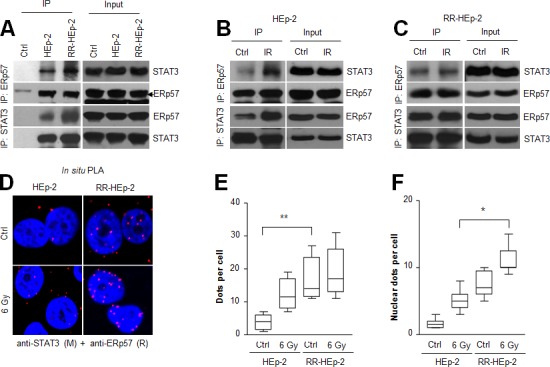
The physical interaction between ERp57 and STAT3 is increased in radioresistant HEp-2 cells (A-C) Indicated cell lysates were immunoprecipitated with anti-ERp57 antibody (upper panel), anti-STAT3 antibody (lower panel) or their respective control immunoglobulin G (IgG: A, Ctrl lane) antibody and immunoblotted with anti-STAT3 or anti-ERp57 antibody. The combined HEp-2 and RR-HEp-2 cell lysates were used as the experimental control (A, Ctrl lane). (B–E) HEp-2 cells or RR-HEp-2 cells were untreated (Ctrl) or treated with 6 Gy radiation (IR) for 12 h. (D-F) The cells were fixed and incubated with mouse anti-STAT3 together with rabbit anti-ERp57, followed by *in situ* PLA analysis. Representative confocal images of cells with PLA-positive signals are shown (D). Dots per cell were counted using CellProfiler (E and F). The data represent typical results and are presented as mean ± standard deviation from 3 independent experiments; **P < 0.01 compared with untreated HEp-2 cells (E) and *P < 0.05 compared with irradiated HEp-2 cells (F).

ERp57 can bind STAT3 [14], a key factor causing resistance in various cancers [24-26]; therefore, we speculated that the molecular interaction between ERp57 and STAT3 may be linked with the radioresistance of laryngeal cancer. To test this possibility, we performed co-immunoprecipitation analysis with ERp57 or STAT3 antibody. Interestingly, co-immunoprecipitation experiments showed that the physical interaction between the two proteins was increased in RR-HEp-2 cells, compared with control cells (Fig. 2A). In addition, the interaction between the two proteins was differentially modulated in the radioresistant cells compared to the control cells, implying that the differential molecular affinity between the two proteins in response to irradiation may be associated with radioresistance (Fig. 2B and C). Co-immunoprecipitation analysis showed that the endogenous level of STAT3 between HEp-2 and RR-HEp-2 cells was similar (Supplementary Fig. 1E). Furthermore, we confirmed this interaction by proximity ligation assay (*in situ* PLA), which can visualize *in vivo* interactions between the two proteins. Consistent with the results of the co-immunoprecipitation experiment, more positive signals indicating interactions between the two proteins were observed in RR-HEp-2 cells than in the control cells, and the positive signals were modulated in response to irradiation (Fig. 2D and E, Supplementary Fig. 2A; negative control experiments). Notably, the interactions in the irradiated cells increased in the nucleus of RR-HEp-2 cells (Fig. 2F), suggesting that increased ERp57-STAT3 interaction is associated with the radioresistance of laryngeal cancer cells. Collectively, our data suggest that the increased interaction between ERp57 and STAT3 in the nucleus is involved with the radioresistance of laryngeal cancer cells.

### ERp57-regulated STAT3 activity in radioresistant laryngeal cancer cells

**Figure 3:**
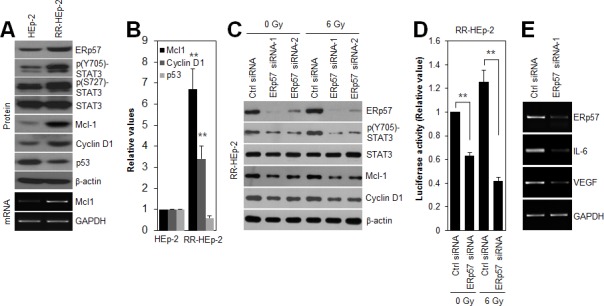
ERp57 modulates STAT3 activity in radioresistant HEp-2 cells (A and B) Lysates of control HEp-2 and RR-HEp-2 were immunoblotted with the indicated antibodies, and the intensities were quantified with ImageJ software. The data represents 3 independent experiments; **P < 0.01 compared with control HEp-2 cells. (C–E) RR-HEp-2 cells were transfected with 100 nM control siRNA, ERp57 siRNA-1, or ERp57 siRNA-2. After 48 h, the cells were treated with 6 Gy radiation. Cells were analyzed by immunoblotting with the indicated antibodies (C). STAT3 activity was determined by STAT3 activity assay. The detailed procedure is described in the Methods section (D). The data represents 3 independent experiments; **P < 0.01 compared with respective control siRNA cells. Gene transcripts, including those of Mcl-1, ERp57, IL-6, and VEGF, were detected by conventional RT-PCR (A and E). GAPDH was used as a loading control. The data represent typical results and are presented as mean ± standard deviation of 3 independent experiments.

To define the role of the ERp57-STAT3 complex in radioresistance of laryngeal cancer cells, we first checked the expression levels of phosphorylated STAT3 and its target genes, Mcl-1, cyclin D1, and p53 in HEp-2 and RR-HEp-2 cells. Notably, phosphorylated STAT3 and its target genes, including Mcl-1 and cyclin D1, were augmented in RR-HEp-2 cells compared to the control cells, whereas p53, a protein that is negatively regulated by STAT3 [27], was downregulated (Fig. 3A), indicating that STAT3 activity is increased in radioresistant laryngeal cancer cells. Out of the STAT3-regulatory genes, Mcl-1, a key anti-apoptotic protein [28], was most significantly upregulated in RR-HEp-2 cells at both the mRNA and protein levels, compared with the corresponding levels in control cells (Fig. 3A and B). To further determine the regulatory effect of ERp57 on STAT3 activity, ERp57 was depleted in RR-HEp-2 cells with siRNAs. Importantly, ERp57 depletion decreased phosphorylated STAT3 and expression of its target genes, Mcl-1 and cyclin D1, in the control and irradiated RR-HEp-2 cells (Fig. 3C). We confirmed this result in two other laryngeal cancer cells (Supplementary Fig. 1C and D). In addition, modulation of STAT3 activity by ERp57 depletion was measured using STAT3 reporter plasmid, which has the STAT3-binding element for luciferase assay [29]. In accordance, STAT3 reporter assay indicated that ERp57 depletion inhibited STAT3 activity compared to the siRNA controls in the control and irradiated RR-HEp-2 cells (Fig. 3D), suggesting that ERp57 enhances STAT3 activity in radioresistant laryngeal cancer cells. Moreover, ERp57 depletion decreased the expression of STAT3-regulated cytokines such as interleukin-6 (IL-6) and vascular endothelial growth factor (VEGF) (Fig. 3E). Thus, our data suggest that increased ERp57-STAT3 interaction enhances STAT3 activity in radioresistant laryngeal cancer cells.

### The ERp57-STAT3-Mcl-1 axis potentiated radioresistance of laryngeal cancer cells

**Figure 4:**
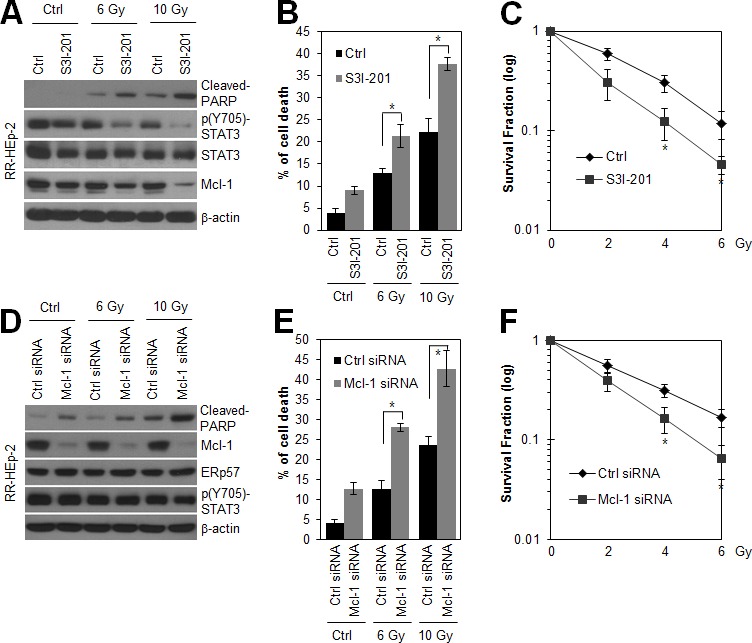
Inhibition of STAT3 activity and depletion of Mcl-1 sensitize radioresistant laryngeal HEp-2 cells (A and B) RR-HEp-2 cells were untreated (Ctrl) or treated with the indicated dose of radiation in the absence (Ctrl) or presence of 100 μM S3I-201 and then incubated for 48 h. (C) RR-HEp-2 cells were treated with each dose of radiation as indicated in the absence or presence of 50 μM S3I-201. High cytotoxicity reduced 100 μM S3I-201 to 50 μM in clonogenic assay. (D-F) RR-HEp-2 cells were transfected with 100 nM control siRNA or Mcl-1 siRNA. After 48 h, the cells were treated with each dose of radiation. (C and F) The clonogenic survival fraction was determined by clonogenic assay. Cell viability was determined by FACScan flow cytometry, and data are presented as the percentage of PI-positive cells (B and E). Cells were analyzed by immunoblotting with the indicated antibodies. β-actin was used as a loading control. The data represent typical results and are presented as mean ± standard deviation of 4 independent experiments; *P < 0.05 compared with irradiated respective control cells.

Because ERp57-STAT3 interaction increased STAT3 activity in radioresistant laryngeal cancer cells, we tested whether inhibition of STAT3 activity sensitizes RR-HEp-2 cells. Notably, S31-201, a direct STAT3 inhibitor, treatment significantly inhibited both STAT3 phosphorylation and Mcl-1 expression, and increased radiation-induced cell death of RR-HEp-2 cells (Fig. 4A and B). Furthermore, S31-201 treatment reduced the survival of RR-HEp-2 cells in response to various doses of radiation (Fig. 4C), indicating that STAT3 activity is essential for the radioresistance of laryngeal cancer cells. Next, we examined whether Mcl-1 downregulation with siRNA modulates the radiation sensitivity of radioresistant laryngeal cancer cells. Similar to the effect of STAT3 inhibition, Mcl-1 depletion also elevated radiation-induced cell death (Fig. 4D and E) and reduced the survival of RR-HEp-2 cells in response to various doses of radiation (Fig. 4F), but it did not affect ERp57 expression and STAT3 phosphorylation (Fig. 4D), indicating that STAT3-Mcl-1 regulation is essential for the radioresistance of laryngeal cancer cells. Taken together, our data suggest that the ERp57-STAT3-Mcl-1 axis confers radioresistance to laryngeal cancer cells.

### *In vivo* evidence of the correlation between ERp57, STAT3, and Mcl-1 in laryngeal cancer tissues

**Figure 5:**
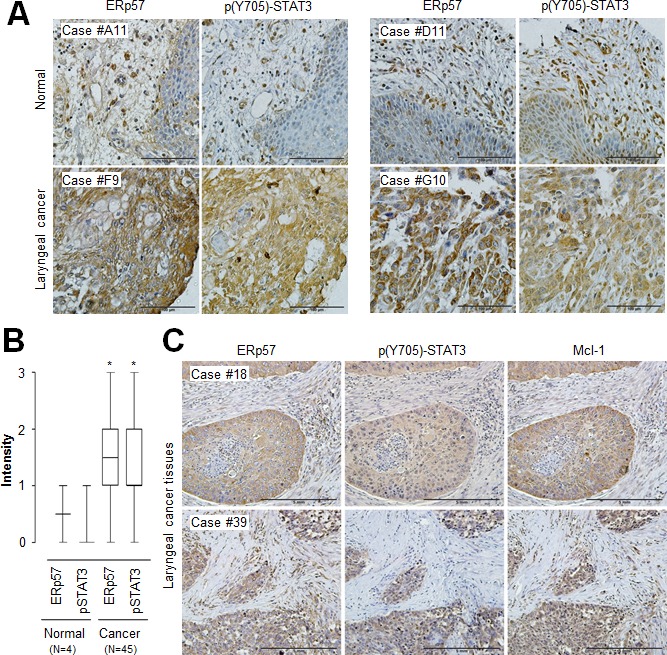
Correlation of ERp57, STAT3, and Mcl-1 expression levels in laryngeal cancer tissues (A) Representative microscopic images of laryngeal cancers and their normal tissue counterpart sections stained with anti-ERp57 antibody (left panels) and anti-phospho-STAT3 (Tyr705) antibody (right panels). Scale bar, 100 μm. (B) Quantification of ERp57 and phospho-STAT3 (Tyr705) staining intensities in laryngeal cancers (*n* = 45) and normal tissues (*n* = 4). Staining intensity was scored as follows: 0, no staining; +1, weak; +2, moderate; and +3, strong. Data are presented as box-and-whisker plots. *P < 0.05 compared with staining intensity of normal tissues. (C) Representative microscopic images of laryngeal cancer tissue sections stained with anti-ERp57 antibody (left panel), anti-phospho-STAT3 (Tyr705) antibody (middle panel), or anti-Mcl-1 antibody (right panel). Scale bar, 5 mm.

To further investigate the physiological relevance of ERp57-STAT3-Mcl-1 regulation in human laryngeal cancer, we first determined the expression of ERp57 and phosphorylated STAT3 using tissue microarrays containing laryngeal cancers and their normal tissue counterparts. We found that both ERp57 and phosphorylated STAT3 were upregulated in laryngeal cancer tissues compared with their normal tissue counterparts (Fig. 5A and B), suggesting that ERp57 expression and STAT3 activation are positively correlated in laryngeal cancer. Since it has been suggested that target gene expression analysis can be a robust indicator of functional STAT3 activation [9], we further evaluated the expression of ERp57 and Mcl-1 using tissue microarrays comprising 59 laryngeal tumor tissues. Notably, ERp57 expression strongly correlated with Mcl-1 expression in laryngeal tumor tissues, based on Spearman's correlation analysis (Table 1). The staining patterns of ERp57 with those of phosphorylated STAT3 or Mcl-1 were also similar in serial sections of the same tissue (Fig. 5C). Taken together, these observations provided *in vivo* evidence for the clinical relevance an ERp57-STAT3-Mcl-1 axis in laryngeal cancer.

**Table 1:** Correlation between ERp57 and Mcl-1 in human laryngeal cancer specimens

	Laryngeal cancer						
	Mcl-1 0	Mcl-1 1+	Mcl-1 2+	Mcl-1 3+	Total (%)	ρ	*P*
ERp57 0	5	1	1	0	7 (11.9%)	0.610	< 0.0001
ERp57 1+	3	17	3	0	23 (39%)		
ERp57 2+	0	4	10	2	16 (27.1%)		
ERp57 3+	0	0	3	10	13 (22%)		
Total	8 (13.6%)	22 (37.3%)	17 (28.8%)	12 (20.3%)	59 (100%)		

ρ indicates Spearman's correlation coefficient. Staining intensity was scored as follows: 0, no staining; +1, weak; +2, moderate; +3, strong.

### *In vivo* interactions between ERp57 and STAT3 were associated with poor prognosis in laryngeal cancer

**Figure 6:**
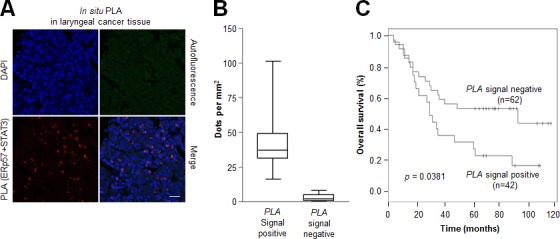
Association between the ERp57-STAT3 interaction and poor prognosis in laryngeal cancer Tissue sections (*n* = 106) were incubated with rabbit anti-ERp57 antibody and mouse anti-STAT3 antibody, followed by *in situ* PLA analysis. (A) Representative confocal images of laryngeal cancers. The red signals represent the complex between ERp57 and STAT3, and nuclei were counterstained with Hoechst 33342 (blue signal). Scale bar, 20 μm. (B) Four different areas were obtained for each sample and 200 - 500 cells were quantified per area (mm^2^) using CellProfiler. Data are presented as box-and-whisker plots. (C) Kaplan-Meier survival curves for samples positive (*n* = 42) for ERp57-STAT3 protein complexes versus those negative (*n* = 62) for ERp57-STAT3 protein complexes for overall survival. *P*-values represent univariate Cox regression analyses.

Due to the association of ERp57-STAT3 interaction with radioresistance of laryngeal cancer, the possibility that the *in vivo* interactions of these two proteins confer a poor prognosis in laryngeal cancer was examined by *in situ* PLA analysis. The detection of positive signals in laryngeal cancer tissues (Fig. 6A, Supplementary Fig. 2B; negative control experiments) indicated the *in vivo* relevance of ERp57-mediated STAT3 regulation. Laryngeal cancer tissues (*n* = 106) with more than 20 red dots per area (mm^2^) were considered as PLA signal positive (Fig. 6B). Importantly, high ERp57-STAT3 interactions correlated with reduced overall survival (*P* = 0.0381; Fig. 6C). Thus, our data suggest that increased ERp57-mediated STAT3 regulation confers a poor prognosis in laryngeal cancer.

## DISCUSSION

Although ERp57 is well known as a stress-responsive protein and chaperone, the regulatory function of ERp57 in tumor resistance and progression has not been well understood. This study provides the first evidence for the novel function of ERp57 in tumor radioresistance. We demonstrated that ERp57 modulated radioresistance of laryngeal cancer cells by directly activating STAT3 and, in turn, triggered increased Mcl-1 expression, thereby contributing to tumor radioresistance of laryngeal cancer cells. The present study also provides *in vivo* evidence that ERp57 expression was tightly associated with the expression of phosphorylated STAT3 or Mcl-1 in laryngeal cancer tissues. Moreover, our findings suggest that high ERp57-mediated STAT3 contributes to poor outcomes in patients with laryngeal cancer in response to radiotherapy, and targeting ERp57-STAT3 is important for enhancing the efficacy of radiotherapy.

In our previous study, using a comparative proteomic approach, we found that ERp57 is increased in radioresistant laryngeal cancer cells [22]. This study further showed that ERp57 expression was regulated by irradiation, and ERp57 depletion sensitized radioresistant laryngeal cancer cells to radiation-induced cell death (Fig. 1). Similar to our observations, recent studies showed that overexpressed ERp57 is a marker for chemoresistance of ovarian cancer, as identified by proteomic analysis [30, 31], and the expression levels of disulfide isomerase family proteins are linked with drug resistance in multiple tumor types [32, 33]. In addition, knockdown of ERp57 enhanced fenretinide-induced apoptosis [34], indicating that increased ERp57 plays a protective role in cancer cells in response to anticancer drugs. This finding suggests that increased ERp57 represents a potential biomarker for radioresistance of laryngeal cancer.

ERp57 has been shown to be induced by various types of stress, such as glucose deprivation or hypoxia, and in certain physiological situations such as neoplastic transformation [14]. However, ERp57 expression was unchanged in response to irradiation in the radioresistant cells. In our previous study, we found that the radioresistant cancer cells are more tolerant than control cells to irradiation owing to the modulation of cellular antioxidant defense proteins such as Mn-SOD, PRDX2, and CLIC1 [22]. Thus, this tolerance mechanism may also be associated with ERp57 expression in the radioresistant cells. ERp57 is localized in many subcellular compartments such as the membrane, cytosol, and nucleus, where it is involved in a variety of functions [14]. Recently, ERp57 was reported to contribute to calreticulin translocation on the cell surface in the process of immunogenic cell death [23, 35], which is an important factor for a favorable outcome in response to chemotherapy treatment of cancer cells. However, our data indicated that ERp57 did not translocate from the cytosol to the cell surface in the irradiated cancer cells, and rather, it was observed in the nucleus in a complex with STAT3 in the radioresistant cells. With regard to the nuclear function of ERp57, it is noteworthy that the nuclear localization of ERp57 is associated with paclitaxel resistance of ovarian cancer cells [31] and positively regulates STAT3 activity in melanoma cells [16]. Thus, nuclear localization of ERp57 might be tightly associated with radioresistance of laryngeal cancer cells.

Several studies have shown that ERp57 interacts with STAT3 in many subcellular compartments. Our data demonstrated that the increased interaction between ERP57 and STAT3 was associated with the radioresistance of laryngeal cancer cells and enhanced STAT3 activity. A previous study showed that ERp57 in the ER lumen negatively regulates STAT3 activation in mouse normal fibroblast cells [17], whereas the ERp57-STAT3 interaction in the cytosol and nucleus contributes to the activation of STAT3 in melanoma cells [15, 16]. Indeed, our *in situ* PLA data indicated that increased ERp57-STAT3 complex association in the nucleus enhanced STAT3 activity in the radioresistant cells. Thus, it is possible that the subcellular compartment of the interaction helps determine STAT3 activity, and dysregulation of this interaction could be associated with pathologic conditions such as cancer. Interestingly, a recent report showed that ERp57 interacts with mTORC1 and positively regulates mTORC1 signaling in the cytosol, and mTORC1 promotes cell proliferation [36]. In addition, another report showed that enhanced ERp57 is linked to oncogenic transformation in normal rat kidney cells [18]. These observations imply that ERp57 acts as an oncogenic protein in the cytosol and nucleus by regulating key oncogenic proteins such as STAT3 and mTORC1.

STAT3 is activated through phosphorylation of tyrosine 705, in response to various cytokines and growth factors such as epidermal growth factor (EGF) and IL-6 [6, 26]. Our previous report showed that growth factor receptors, including EGFR and HER-2, are highly upregulated in RR-HEp-2 cells compared to the control cells [22], suggesting that activation of STAT3 in the radioresistant cells may be associated with the upregulation of growth factor receptors, including EGFR and HER-2. In addition, STAT3 activity is modulated by endogenous regulatory proteins such as SOCS-3 and PIAS [26]. Given that other groups and we have shown that ERp57 modulates transcriptional activity of STAT3 [15, 16], it is possible that ERp57 is an endogenous regulatory protein of STAT3. Notably, we also showed that ERp57 depletion decreased IL-6, a major upstream activator of STAT3 [8, 26], in radioresistant laryngeal cancer cells, suggesting that ERp57 regulates STAT3 activity by modulating not only STAT3 activity directly but also IL-6 expression, thereby creating a positive feedback loop. Furthermore, we found that the STAT3-Mcl-1 axis was associated with ERp57-mediated radioresistance. Mcl-1 is a key factor in the resistance of a variety cancers to radiation and chemotherapy [28]. For example, Mcl-1 down-regulation sensitizes cancer cells to anticancer drugs [37, 38]. Consistent with these observations, our data indicated that targeting Mcl-1 downregulation significantly sensitized radioresistant laryngeal cancer cells to irradiation. Therefore, the ERp57-STAT3-Mcl-1 axis might be essential for radiation resistance of laryngeal cancer cells.

ERp57 is upregulated in breast, uterus, lung, and stomach tumors, and it is downregulated in colon cancer [39]. In addition, a recent report showed that ERp57 is overexpressed in cervical cancer and serves as an independent prognostic marker [19]. Similar to these observations, our immunohistochemical analysis demonstrated that ERp57 was overexpressed in patients with laryngeal cancer. Consistent with our observation, bioinformatic analysis using the Oncomine database (www.oncomine.org) indicated that ERp57 is highly expressed in head and neck cancer (Supplementary Figure. 2), which supports that ERp57 is associated with the development of laryngeal cancer. However, other reports showed that loss of ERp57 is associated with poor prognosis in gastric cancer [21]. Thus, this discrepancy may be due to the difference in tumor types. In addition, we showed that ERp57 expression was tightly associated with phosphorylated STAT3 and Mcl-1 expression in laryngeal cancer tissues. Increased phosphorylated STAT3 and Mcl-1 have been often associated with poor prognosis and poor outcome response to chemo- and radiotherapy in various tumors, including head and neck cancer, and inhibition of STAT3 is known to block cancer cell proliferation [7, 9]. Moreover, ERp57 has a role in conferring resistance to apoptosis under hypoxia and endoplasmic reticulum stress [40], and it is involved in bone metastasis of breast cancer [20]. In the present study, we showed that increased ERp57-STAT3 complex association was correlated with poor prognosis of laryngeal cancer. Collectively, these observations indicate that ERp57 may be a clinical marker of radiation resistance and poor prognosis in laryngeal cancer.

In conclusion, this study showed that ERp57 potentiated radiation resistance of laryngeal cancer via ERp57-STAT3-Mcl-1 axis regulation. Our work provides evidence of an oncogenic role of ERp57 in regulating STAT3 activation in tumor development and resistance.

## METHODS

### Cell lines and treatment

Human laryngeal squamous cell carcinoma HEp-2 cells were purchased from the American Type Culture Collection (ATCC, Manassas, VA) and human laryngeal squamous cell carcinoma SNU899 and SNU1076 cells were obtained from the Korean Cell Line Bank (Seoul, Korea). Cells were grown in DMEM supplemented with 10% fetal bovine serum (HyClone, South Logan, UT) and penicillin/streptomycin at 37°C in a humidified 5% CO_2_ incubator. Radioresistant laryngeal cancer HEp-2 (RR-HEp-2) cells were established as previously described [22, 41]. The cells were irradiated using a ^137^cesium (Cs) ray source (Atomic Energy of Canada Ltd., Mississauga, Canada) at a dose rate of 3.81 Gy/min. S3I-201 (100 μM; EMD Millipore, Billerica, MA) was used to inhibit STAT3 activity.

### Clonogenic assay

Cell survival after irradiation was determined by a clonogenic assay as previously described [22]. Briefly, cells were treated with various doses of radiation, and then, the irradiated cells were seeded in triplicate in 60-mm tissue culture dishes at various densities (200 cells for control, 400 cells for 2 Gy, 1500 cells for 4 Gy, and 3000 cells for 6 Gy). After 10–14 d, the colonies were fixed with methanol and stained with a Trypan blue solution. Only colonies containing more than 50 cells were counted as surviving colonies.

### RNA interference

The siRNAs were synthesized at Genolution Pharmaceuticals Inc. (Seoul, Korea). The sequences of siRNAs against human ERp57, STAT3, and Mcl-1 were as follows: ERp57-#1, 5′-GGACAAGACUGUGGCAUAU-3′; ERp57-#2, 5′-GGGCAAGGACUUACUUAUU-3′; STAT3, 5′-CCAACGACCUGCAGCAAUA-3′; and Mcl-1, 5′-CCCGCCGAAUUCAUUAAUUUA-3′. A non-targeting siRNA (Genolution Pharmaceuticals Inc.) was used as a negative control. Transfection of siRNA was performed using Lipofectamine 2000 (Invitrogen, Carlsbad, CA), according to the manufacturer's protocol.

### Western blot analysis

Western blotting was performed as described previously [22, 42]. Briefly, proteins were separated by SDS-polyacrylamide gel electrophoresis, transferred to a nitrocellulose membrane, and detected using specific antibodies. The following antibodies were used: rabbit polyclonal anti-phospho-STAT3 (Tyr705), anti-phospho-STAT3 (Ser727), and anti-cleaved-PARP (Asp214) from Cell Signaling Technology (Beverly, MA) as well as mouse monoclonal anti-cyclin D1, anti-Mcl-1, anti-ERp57, and anti-STAT3 from Santa Cruz Biotechnology Inc. (Santa Cruz, CA) and anti-p53 and anti-β-actin from Sigma. Blots were developed using HRP-conjugated secondary antibody and enhanced chemiluminescence detection system (Amersham Life Science, Piscataway, NJ).

### Reverse transcription polymerase chain reaction (RT-PCR)

RT-PCR was performed following a previously described protocol [22]. Briefly, total RNA isolated using STAT-60 (Tel-Test B, Inc., Friendswood, TX) was reverse-transcribed with ImProm-II™ reverse transcription system (Promega, Madison, WI). The following PCR primers were employed for conventional PCR: ERp57, sense 5′-CCTGGTGTGGACACTGCAAG-3′ and antisense 5′-CCCTCAAGTTGCTGGCTGCT-3′; IL-6, sense 5′-CCTGAGAAAGGAGACATGTAACAAGA-3′ and antisense 5′-GGCAAGTCTCCTCATTGAATCC-3′; Mcl-1, sense 5′-ATCTCTCGGTACCTTCGGGAG-3′ and antisense 5′-ACCAGCTCCTACTCCAGCAAC-3′; VEGF, sense 5′-CGAAGTGGTGAAGTTCATGGATG-3′ and antisense 5′-TTCTGTATCAGTCTTTCCTGGTGAG-3′; and glyceraldehyde 3-phosphate dehydrogenase (GAPDH), sense 5′-catctctgccccctctgctga-3′ and antisense 5′-ggatgaccttgcccacagcct-3′.

### STAT3 transcriptional activity

STAT3 transcriptional activity was determined as previously described [29]. Briefly, the cells were co-transfected with 21pSTAT3-TA-Luc and control siRNA or ERp57 siRNA for 48 h using Lipofectamine 2000 (Invitrogen) and then untreated or irradiated with 6 Gy. After 24 h, the cells were harvested using passive lysis buffer, and luciferase activity was evaluated using the Dual Luciferase Reporter Assay Kit (Promega) on a Wallac Victor2 plate reader (Perkin Elmer Corp., Norwalk, CT).

### Cell death analysis

Cell death analysis was performed as previously described [22]. Briefly, cells were trypsinized, washed, and then incubated with propidium iodide (5 μg/mL) for 10 min at room temperature, and the cells were analyzed with the FACScan flow cytometer (Becton Dickson, Franklin Lakes, NJ).

### Immunofluorescence assay

Immunofluorescence assay was performed as previously described [43]. Briefly, cells were fixed with 4% paraformaldehyde, permeabilized, and blocked with 0.2% Triton X-100 and 5% fetal calf serum in PBS. The fixed cells were consecutively incubated with mouse anti-ERp57 antibody (1:200 dilution; Santa Cruz Biotechnology Inc.) and anti-mouse Alexa-488 (Molecular Probes, Eugene, OR). F-actin was detected with Alexa Fluor 568-conjugated phalloidin (Invitrogen). Slides were mounted in a medium containing DAPI, and images were then obtained using a confocal laser-scanning microscope (LSM 710; Carl Zeiss, Inc., Oberkochen, Germany).

### Immunohistochemistry

Human tissue microarrays were purchased from SuperBioChips (Cat Number: CH3; Seoul, Korea) and AccuMax (Cat Number: A220; Seoul, Korea). Immunohistochemistry was performed as previously described [43, 44]. Briefly, immunohistochemical staining was performed with an anti-ERp57 rabbit polyclonal antibody (1:100 dilution; Santa Cruz Biotechnology Inc.), anti-Mcl-1 rabbit polyclonal antibody (1:100 dilution; Santa Cruz Biotechnology Inc.), or anti-phospho-STAT3 (Tyr705) rabbit polyclonal antibody (1:50 dilution; GeneTex, Irvine, CA). Immunostaining was performed with the avidin-biotin-peroxidase method, according to the manufacturer's instructions (Invitrogen). Staining intensity was scored as follows: 0 (no visible staining), 1+ (faint staining), 2+ (moderate staining), and 3+ (strong staining).

### Immunoprecipitation

Immunoprecipitation was performed as previously described [45]. Briefly, cells were lysed with NP-40 lysis buffer, and the lysates were then precipitated with a negative control mouse antibody (Santa Cruz Biotechnology Inc.) or a mouse monoclonal antibody against ERp57 (Santa Cruz Biotechnology Inc.). Immune complexes were collected using protein G-Sepharose and washed 3 times, and SDS sample buffer was then added. The samples were size-fractionated by electrophoresis and detected using immunoblotting.

### *In situ* proximity ligation assay (PLA)

*In situ* PLA was performed as previously described [43]. Briefly, the paraformaldehyde-fixed cells were permeabilized with 0.2% Triton X-100, washed, and blocked with blocking solution (Olink Bioscience, Uppsala, Sweden). Antigen-retrieved cancer tissues (SuperBioChips) were incubated with 3% hydrogen peroxide, washed, and blocked with blocking solution. A mouse monoclonal anti-ERp57 antibody (Santa Cruz Biotechnology Inc.; 1:200) and a rabbit polyclonal anti-STAT3 antibody (Santa Cruz Biotechnology Inc.; 1:200) were used for the PLA. The assay was performed according to the manufacturer's protocol, using the Duolink Detection Kit with a pair of nucleotide-labeled secondary antibodies (Olink Bioscience). Amplified PLA signals were analyzed using confocal microscopy and quantified using CellProfiler software (www.cellprofiler.org).

### Statistical analysis

A two-tailed Student's *t*-test was performed to analyze statistical differences between groups. The correlation between ERp57 and Mcl-1 immunointensity was analyzed using a Spearman's rank correlation test. The Kaplan-Meier method was used for the survival analysis and the statistical significance was analyzed by the Log-rank test. A Cox proportional hazards model was used to evaluate the differences between negative and positive PLA signals. *P* < 0.05 was considered statistically significant.

## SUPPLEMENTARY MATERIAL FIGURES



## References

[1] Parkin DM, Bray F, Ferlay J, Pisani P (2001). Estimating the world cancer burden: Globocan 2000. International journal of cancer. Journal international du cancer.

[2] Lefebvre JL (2006). Laryngeal preservation in head and neck cancer: multidisciplinary approach. The lancet oncology.

[3] Nix PA, Greenman J, Cawkwell L, Stafford N (2004). Radioresistant laryngeal cancer: beyond the TNM stage. Clinical otolaryngology and allied sciences.

[4] Kim JJ, Tannock IF (2005). Repopulation of cancer cells during therapy: an important cause of treatment failure. Nature reviews. Cancer.

[5] Begg AC, Stewart FA, Vens C (2011). Strategies to improve radiotherapy with targeted drugs. Nature reviews. Cancer.

[6] Yu H, Jove R (2004). The STATs of cancer--new molecular targets come of age. Nature reviews. Cancer.

[7] Song JI, Grandis JR (2000). STAT signaling in head and neck cancer. Oncogene.

[8] Yu H, Pardoll D, Jove R (2009). STATs in cancer inflammation and immunity: a leading role for STAT3. Nature reviews. Cancer.

[9] Frank DA (2013). Transcription factor STAT3 as a prognostic marker and therapeutic target in cancer. Journal of clinical oncology : official journal of the American Society of Clinical Oncology.

[10] Gao L, Li F, Dong B, Zhang J, Rao Y, Cong Y, Mao B, Chen X (2010). Inhibition of STAT3 and ErbB2 suppresses tumor growth, enhances radiosensitivity, and induces mitochondria-dependent apoptosis in glioma cells. International journal of radiation oncology, biology, physics.

[11] Li X, Wang H, Lu X, Di B (2010). STAT3 blockade with shRNA enhances radiosensitivity in Hep-2 human laryngeal squamous carcinoma cells. Oncology reports.

[12] Bournazou E, Bromberg J (2013). Targeting the tumor microenvironment: JAK-STAT3 signaling. JAK-STAT.

[13] Noman MZ, Buart S, Van Pelt J, Richon C, Hasmim M, Leleu N, Suchorska WM, Jalil A, Lecluse Y, El Hage F, Giuliani M, Pichon C, Azzarone B (2009). The cooperative induction of hypoxia-inducible factor-1 alpha and STAT3 during hypoxia induced an impairment of tumor susceptibility to CTL-mediated cell lysis. Journal of immunology.

[14] Turano C, Gaucci E, Grillo C, Chichiarelli S (2011). ERp57/GRP58: a protein with multiple functions. Cellular & molecular biology letters.

[15] Eufemi M, Coppari S, Altieri F, Grillo C, Ferraro A, Turano C (2004). ERp57 is present in STAT3-DNA complexes. Biochemical and biophysical research communications.

[16] Chichiarelli S, Gaucci E, Ferraro A, Grillo C, Altieri F, Cocchiola R, Arcangeli V, Turano C, Eufemi M (2010). Role of ERp57 in the signaling and transcriptional activity of STAT3 in a melanoma cell line. Archives of biochemistry and biophysics.

[17] Coe H, Jung J, Groenendyk J, Prins D, Michalak M (2010). ERp57 modulates STAT3 signaling from the lumen of the endoplasmic reticulum. The Journal of biological chemistry.

[18] Hirano N, Shibasaki F, Sakai R, Tanaka T, Nishida J, Yazaki Y, Takenawa T, Hirai H (1995). Molecular cloning of the human glucose-regulated protein ERp57/GRP58, a thiol-dependent reductase. Identification of its secretory form and inducible expression by the oncogenic transformation. European journal of biochemistry / FEBS.

[19] Liao CJ, Wu TI, Huang YH, Chang TC, Wang CS, Tsai MM, Lai CH, Liang Y, Jung SM, Lin KH (2011). Glucose-regulated protein 58 modulates cell invasiveness and serves as a prognostic marker for cervical cancer. Cancer science.

[20] Santana-Codina N, Carretero R, Sanz-Pamplona R, Cabrera T, Guney E, Oliva B, Clezardin P, Olarte OE, Loza-Alvarez P, Mendez-Lucas A, Perales JC, Sierra A (2013). A transcriptome-proteome integrated network identifies endoplasmic reticulum thiol oxidoreductase (ERp57) as a hub that mediates bone metastasis. Molecular &; cellular proteomics : MCP.

[21] Leys CM, Nomura S, LaFleur BJ, Ferrone S, Kaminishi M, Montgomery E, Goldenring JR (2007). Expression and prognostic significance of prothymosin-alpha and ERp57 in human gastric cancer. Surgery.

[22] Kim JS, Chang JW, Yun HS, Yang KM, Hong EH, Kim DH, Um HD, Lee KH, Lee SJ, Hwang SG (2010). Chloride intracellular channel 1 identified using proteomic analysis plays an important role in the radiosensitivity of HEp-2 cells via reactive oxygen species production. Proteomics.

[23] Panaretakis T, Joza N, Modjtahedi N, Tesniere A, Vitale I, Durchschlag M, Fimia GM, Kepp O, Piacentini M, Froehlich KU, van Endert P, Zitvogel L, Madeo F (2008). The co-translocation of ERp57 and calreticulin determines the immunogenicity of cell death. Cell death and differentiation.

[24] Catlett-Falcone R, Landowski TH, Oshiro MM, Turkson J, Levitzki A, Savino R, Ciliberto G, Moscinski L, Fernandez-Luna JL, Nunez G, Dalton WS, Jove R (1999). Constitutive activation of Stat3 signaling confers resistance to apoptosis in human U266 myeloma cells. Immunity.

[25] Gritsko T, Williams A, Turkson J, Kaneko S, Bowman T, Huang M, Nam S, Eweis I, Diaz N, Sullivan D, Yoder S, Enkemann S, Eschrich S (2006). Persistent activation of stat3 signaling induces survivin gene expression and confers resistance to apoptosis in human breast cancer cells. Clinical cancer research : an official journal of the American Association for Cancer Research.

[26] Brantley EC, Benveniste EN (2008). Signal transducer and activator of transcription-3: a molecular hub for signaling pathways in gliomas. Molecular cancer research : MCR.

[27] Niu G, Wright KL, Ma Y, Wright GM, Huang M, Irby R, Briggs J, Karras J, Cress WD, Pardoll D, Jove R, Chen J, Yu H (2005). Role of Stat3 in regulating p53 expression and function. Molecular and cellular biology.

[28] Akgul C (2009). Mcl-1 is a potential therapeutic target in multiple types of cancer. Cellular and molecular life sciences : CMLS.

[29] Shin DS, Kim HN, Shin KD, Yoon YJ, Kim SJ, Han DC, Kwon BM (2009). Cryptotanshinone inhibits constitutive signal transducer and activator of transcription 3 function through blocking the dimerization in DU145 prostate cancer cells. Cancer research.

[30] Cicchillitti L, Di Michele M, Urbani A, Ferlini C, Donat MB, Scambia G, Rotilio D (2009). Comparative proteomic analysis of paclitaxel sensitive A2780 epithelial ovarian cancer cell line and its resistant counterpart A2780TC1 by 2D-DIGE: the role of ERp57. Journal of proteome research.

[31] Cicchillitti L, Della Corte A, Di Michele M, Donati MB, Rotilio D, Scambia G (2010). Characterisation of a multimeric protein complex associated with ERp57 within the nucleus in paclitaxel-sensitive and -resistant epithelial ovarian cancer cells: the involvement of specific conformational states of beta-actin. International journal of oncology.

[32] Lee AS (2001). The glucose-regulated proteins: stress induction and clinical applications. Trends in biochemical sciences.

[33] Lovat PE, Corazzari M, Armstrong JL, Martin S, Pagliarini V, Hill D, Brown AM, Piacentini M, Birch-Machin MA, Redfern CP (2008). Increasing melanoma cell death using inhibitors of protein disulfide isomerases to abrogate survival responses to endoplasmic reticulum stress. Cancer research.

[34] Corazzari M, Lovat PE, Armstrong JL, Fimia GM, Hill DS, Birch-Machin M, Redfern CP, Piacentini M (2007). Targeting homeostatic mechanisms of endoplasmic reticulum stress to increase susceptibility of cancer cells to fenretinide-induced apoptosis: the role of stress proteins ERdj5 and ERp57. British journal of cancer.

[35] Panaretakis T, Kepp O, Brockmeier U, Tesniere A, Bjorklund AC, Chapman DC, Durchschlag M, Joza N, Pierron G, van Endert P, Yuan J, Zitvogel L, Madeo F (2009). Mechanisms of pre-apoptotic calreticulin exposure in immunogenic cell death. The EMBO journal.

[36] Ramirez-Rangel I, Bracho-Valdes I, Vazquez-Macias A, Carretero-Ortega J, Reyes-Cruz G, Vazquez-Prado J (2011). Regulation of mTORC1 complex assembly and signaling by GRp58/ERp57. Molecular and cellular biology.

[37] Chen S, Dai Y, Harada H, Dent P, Grant S (2007). Mcl-1 down-regulation potentiates ABT-737 lethality by cooperatively inducing Bak activation and Bax translocation. Cancer research.

[38] Ding Q, Huo L, Yang JY, Xia W, Wei Y, Liao Y, Chang CJ, Yang Y, Lai CC, Lee DF, Yen CJ, Chen YJ, Hsu JM (2008). Down-regulation of myeloid cell leukemia-1 through inhibiting Erk/Pin 1 pathway by sorafenib facilitates chemosensitization in breast cancer. Cancer research.

[39] Celli CM, Jaiswal AK (2003). Role of GRP58 in mitomycin C-induced DNA cross-linking. Cancer research.

[40] Ni M, Lee AS (2007). ER chaperones in mammalian development and human diseases. FEBS letters.

[41] Kim JS, Yun HS, Um HD, Park JK, Lee KH, Kang CM, Lee SJ, Hwang SG (2012). Identification of inositol polyphosphate 4-phosphatase type II as a novel tumor resistance biomarker in human laryngeal cancer HEp-2 cells. Cancer Biol Ther.

[42] Kim JS, Chang JW, Park JK, Hwang SG (2012). Increased aldehyde reductase expression mediates acquired radioresistance of laryngeal cancer cells via modulating p53. Cancer Biol Ther.

[43] Kim JS, Kim EJ, Oh JS, Park IC, Hwang SG (2013). CIP2A modulates cell-cycle progression in human cancer cells by regulating the stability and activity of Plk1. Cancer research.

[44] Min JW, Kim KI, Kim HA, Kim EK, Noh WC, Jeon HB, Cho DH, Oh JS, Park IC, Hwang SG, Kim JS (2013). INPP4B-mediated tumor resistance is associated with modulation of glucose metabolism via hexokinase 2 regulation in laryngeal cancer cells. Biochemical and biophysical research communications.

[45] Kim JS, Park ZY, Yoo YJ, Yu SS, Chun JS (2005). p38 kinase mediates nitric oxide-induced apoptosis of chondrocytes through the inhibition of protein kinase C zeta by blocking autophosphorylation. Cell Death Differ.

