# Supplementary material for: ERp57 modulates STAT3 activity in radioresistant laryngeal cancer cells and serves as a prognostic marker for laryngeal cancer
Source: Oncotarget. 2015 Jan 8;6(5):2654–66. doi: 10.18632/oncotarget.3042 (PMC4413608; doi:10.18632/oncotarget.3042)
Supplement: Supplementary file 1 [file oncotarget-06-2654-s001.pdf]

ERp57 modulates STAT3 activity in radioresistant laryngeal cancer cells and serves as a prognostic marker for laryngeal cancer

Supplementary Material

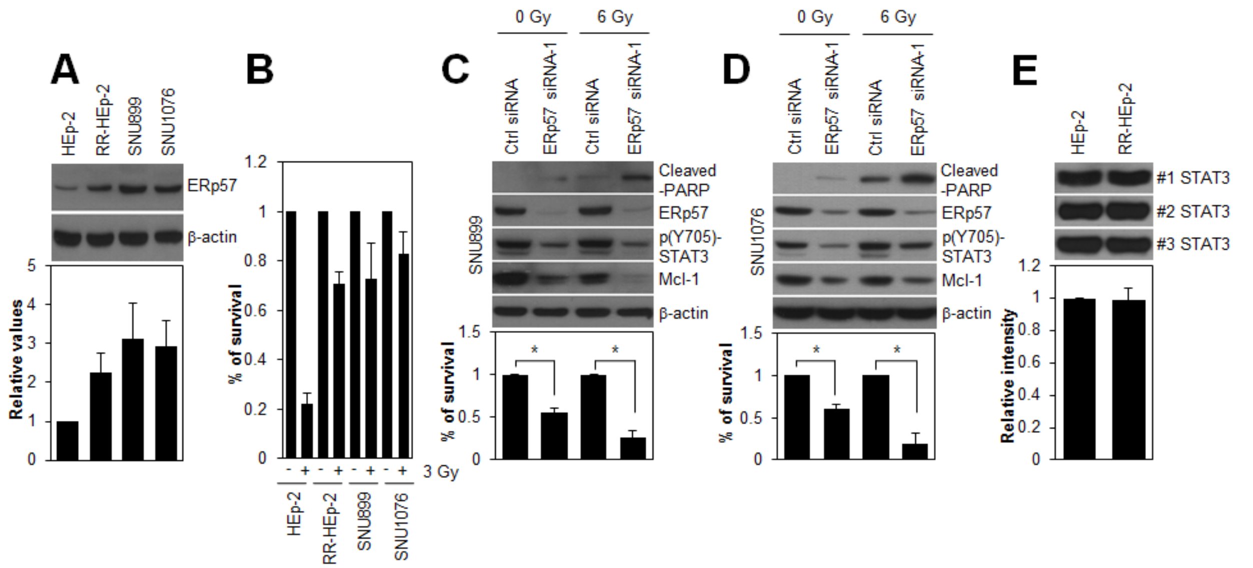

Supplementary Figure 1: ERp57-STAT3 regulation is associated with radioresistance in other laryngeal cancer cell lines. (A) Lysates of HEp-2, RR-HEp-2, SNU899, and SNU1076 cells were immunoblotted with anti-ERp57 antibody. (B) HEp-2, RR-HEp-2, SNU899, and SNU1076 cells were untreated or treated with 3 Gy radiation and colony formation was quantified by automatic colony counter. (C and D) SNU899 or SNU1076 cells were transfected with 100 nM control siRNA or ERp57 siRNA-1. After 48 h, the cells were treated with 6 Gy (upper panel) or 3 Gy (lower panel) radiation. Cells were analyzed by immunoblotting with the indicated antibodies (upper panel) or colony formation was quantified by automatic colony counter (lower panel). (E) HEp-2 and RR-HEp-2 cell lysates from 3 independent experiments were immunoblotted with anti-STAT3 antibody. Expression levels of ERp57 (A) or STAT3 (E) were quantified using ImageJ software. *$P < 0.05$ with respect to control siRNA cells (C and D).

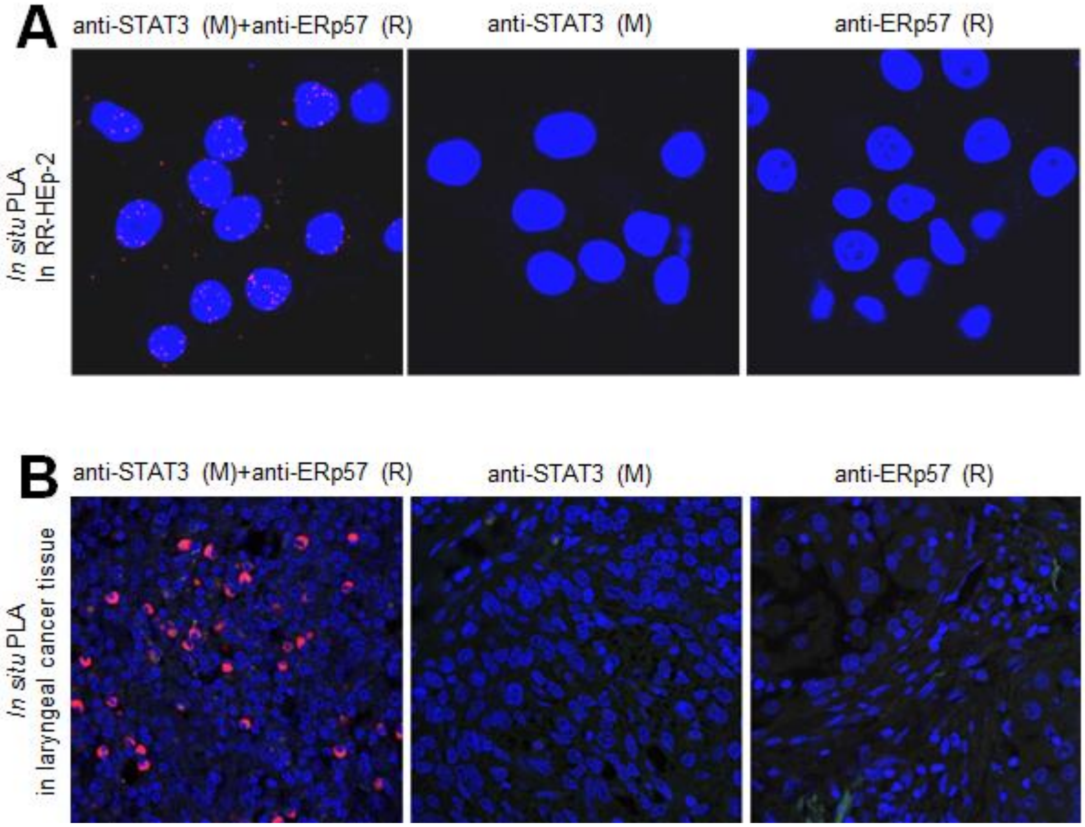

Supplementary Figure 2: Negative control of *in situ* PLA analysis in laryngeal cancer cells and tissues. (A) RR-Hep-2 cells or (B) laryngeal cancer tissues were fixed and incubated with mouse anti-STAT3 and rabbit anti-ERp57 (left), mouse anti-STAT3 and control rabbit IgG (middle), or rabbit anti-ERp57 and control mouse IgG (right), followed by *in situ* PLA analysis. Representative confocal images of cells or tissues with PLA-positive signals.

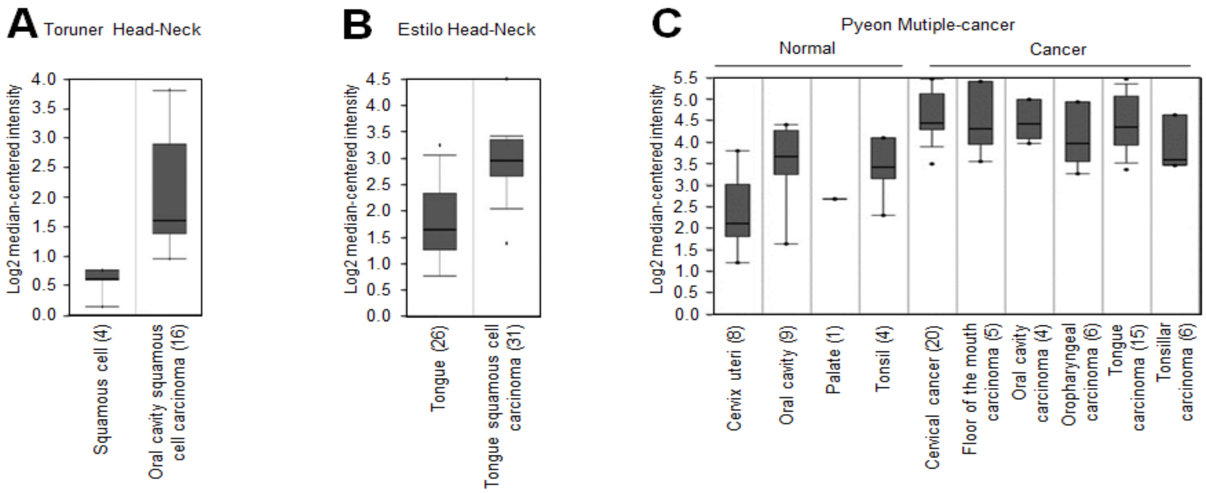

Supplementary Figure 3: ERp57 expression in cancer and normal tissues. Oncomine database (www.oncomine.org) was queried for the expression of ERp57 in the available datasets based on the cancer or normal status (Threshold *P*-value: 1E-4 and fold change: 2). (A and B) ERp57 expression in normal and head and neck cancer tissues in the Toruner dataset (A) and Estilo dataset (B). (C) ERp57 expression in normal and cancer tissues in the Pyeon dataset.
